# Emotions and team communication in the operating room: a scoping review

**DOI:** 10.1080/10872981.2023.2194508

**Published:** 2023-03-30

**Authors:** Henrietta Lee, Robyn Woodward-Kron, Alan Merry, Jennifer Weller

**Affiliations:** a Centre for Medical and Health Sciences Education, School of Medicine, University of Auckland, Grafton,Auckland 1023, New Zealand; b Department of Anaesthesiology, School of Medicine, The University of Auckland, Grafton, Auckland 1023, New Zealand; c Department of Medical Education, Melbourne Medical School, The University of Melbourne, Parkville, VIC 3010, Australia; d Honorary Consultant, Department of Anaesthesia and Perioperative Care, Auckland City Hospital, Park Rd, Grafton, Auckland 1023, New Zealand

**Keywords:** Communication, emotions, operating theatre, interprofessional, scoping review

## Abstract

Training in healthcare team communication has largely focused on strategies to improve information transfer with less focus on interpersonal dynamics and emotional aspects of communication. The Operating Room (OR) may be one of the most emotionally charged hospital environments, and is one requiring excellent team communications. We aimed to identify literature reporting on the emotional aspects of OR team communication. Our research questions were: what are the triggers in the environment that provoke an emotional response affecting communication, and what are the emotional responses to communication between OR team members; and how do these emotional aspects of communication affect the function of the OR team? We undertook a Scoping Review of literature across relevant databases following published guidelines, and narrative synthesis of the identified studies. From the 10 included studies we identified three themes: (1) Emotional experiences in the OR and their contributors; (2) Effects of emotional experiences on team communication; and (3) Solutions to manage the emotional experiences in the OR. Theme 1 sub-themes were: (1) Range of emotions experienced in the OR; (2) Hierarchical culture and (3) Leadership expectations as contributors to negative emotions. The OR is an emotionally charged environment. The hierarchical culture can inhibit staff from speaking up, and failure of leaders to meet team expectations, e.g., through appropriate and timely communication, may cause frustration and stress. The consequences of emotions include poor team dynamics, ineffective communication and potential negative impact on patient care. Few studies described strategies to manage emotions in the OR. The studies reviewed describe an environment where emotions can run high, affecting interpersonal communications, team function and patient care. The few identified studies relevant to our research questions demonstrate a need to better understand the emotional aspects of OR team communication and the effectiveness of interventions to improve these.

## Introduction

Effective communication among multidisciplinary health professionals in a surgical team is necessary to deliver high-quality care to patients and minimise patient harm [1,2]. Many researchers have designed interventions to improve team communication in the operating room (OR), such as briefing [3,4]; closed-loop communication [5,6] and techniques to encourage junior staff to speak up [7,8]. While these interventions may not have been widely adopted in the workplace [9–11], there is evidence that, when used in the OR, they may lead to: improved perception of team communication [3]; improved transfer of information [4,5]; increased quality of communication [12]; more structured communications for handover of patient information [4,5,13–15]; and improved ability to speak up with concerns [8].

However, communication is more than an act of transfer of information, and clinical educators have perhaps spent less time on the interpersonal dimensions of communication. For example, stress, anxiety or frustration may influence how a clinician communicates, and how that communication is perceived and responded to by others. A communication may invoke an emotional response in the receiver, or the team, such as distress, anxiety, or defensiveness. These emotional responses may be accompanied by a physiological response such as increased heart rate, sweating or tremor. Such responses may affect subsequent team interactions, and ensuing team function and may potentially impact on patient care.

According to the linguist Halliday [16], the interpersonal function of language expresses the role of each speaker in an interaction, including interpersonal dynamics between speakers and emotional cues. The choice of words and phrases used in an interaction between speakers will likely reflect the context of the communication, including the interpersonal dynamics of the speakers involved: for example, a surgeon calling an anaesthetist ‘Anaesthesia’ rather than by name may be a hierarchical dynamic which suggests that the leader doesn’t need to know the names of their team members. Extensive use of imperatives when making requests, for example, ‘get the next patient,’ may negatively affect the interactional dynamics whereas more polite phrasing, such as ‘are we ready to fetch the next patient?’, ‘please could you’, ‘would you mind’, ‘thank you’ may have a more positive impact. The speaker’s intonation may also have an impact [16].

In the context of hospital ORs, Lingard et al. [17] noted that non-verbal cues, tone of voice, or use of repetition and emphasis can indicate signs of tension [17]. When surgical team members are under stress, their communication patterns may change and this has implications for other team members, including both their actions and their sense of well-being. In another study, anaesthetists rated communication with surgeons and other hospital staff as one of the greatest occupational stressors [18]. Stress may trigger negative emotions, and both stress and negative emotions have detrimental effects on team communication, job satisfaction and well-being [18]. In an interview study of senior OR staff, participants described how a team member raising concerns about their actions could provoke a negative and unhelpful response, which in turn could affect the rest of the healthcare team, potentially limiting effectiveness of patient care [19].

Thus it seems that emotions experienced by health professionals in the OR work environment are important, and may affect the quality of team communication, team performance and team-member well-being [20] and potentially impacting on the effectiveness of team-building, interprofessional communication and patient care.

The purpose of this scoping review was to identify and synthesise the current state of knowledge regarding the emotional aspects of team communication in the complex and fast-moving OR environment. For the purpose of this review, we used the MerriamWebster’s definition of emotion as: *‘A conscious mental reaction (such as anger or fear) subjectively experienced as strong feeling usually directed toward a specific object and typically accompanied by physiological and behavioural changes in the body.’* (URL: https://www.merriam-webster.com/dictionary/emotions). We adapted this definition to the context of team communication and defined the emotional aspects of communication as: the conscious mental reaction subjectively experienced as strong feeling that may influence how one person communicates to another, or responds to another’s communication. We included in this emotional response both verbal and non-verbal behaviours (e.g., shouting or stomping around the room).

The specific research questions for our scoping review were:

1. What are the triggers in the environment that provoke an emotional response affecting communication, and what are the emotional responses to communication between the health professionals who work in the OR?
2. How do these emotional aspects of communication affect the function of the OR team?

## Materials and methods

We undertook a scoping review [21] and narrative synthesis of the literature to examine the current state of knowledge regarding the emotional aspects of team communication in the context of the OR. This review method was chosen because of the exploratory nature of the review and the inclusion of studies with heterogeneous designs and methodology. This review was prepared in accordance with the Preferred Reporting Items for Systematic reviews and Meta-Analyses extension for Scoping Reviews (PRISMA-ScR) Checklist guidelines [22]. The term *team communication* refers to all verbal and non-verbal exchanges between all members of the healthcare team in the OR treating the patient.

### Search strategy

A keyword search was conducted on the following databases: PubMed, Medline (OVID), Embase, CINAHL. To define the search terms, we reviewed the Medical Subject Headings (MeSH) database and consulted a librarian at the Faculty of Medical and Health Sciences, University of Auckland. The following search terms were used: [emotions OR stress OR happiness OR anxiety OR frustration OR aggression OR well-being OR quality of life OR psychological safety] AND communication AND [operating room OR operating theatre OR surgery]. We included the above range of alternative terms to capture the literature relevant to different topics in the healthcare literature where emotions or emotional responses were potentially discussed. As we were seeking interpersonal communication in the OR team, we selected terms that referred to the OR environment rather than specific disciplinary groups. This search was conducted on 29 May 2019 and repeated on 18 September 2021 and included all literature up to the second week of September 2021. No limit was used on the start date of the search period.

Depending on the search requirements of each database, the search terms were modified to take into account alternative spellings (e.g., theatre, theater), synonyms or variations of the same search term (e.g., operating room, operating theatre), or truncated to include different suffixes attached to the same word root (e.g., surgeries, surgery, surgical). Phrases such as ‘quality of life’ or ‘well being’ or ‘psychological safety’, both with and without hyphen variations, were searched together as exact phrases. For all databases, the filters or limits used in the searches were human participants and article published in the English language. To maximise inclusion of articles relevant to the review, all types of studies (e.g., qualitative, quantitative, mixed methods, reviews, editorials) were included in the review.

### Inclusion and Exclusion Criteria

An article was included if:

The research reported the direct emotions of participants in the context of communication between two or more health professionals within the hospital OR environment. Specifically, the article’s results and findings needed to include either (1) direct reports of participants’ feelings or (2) reports of verbal or non-verbal behaviour suggesting an emotional response

(e.g. barking commands, stomping around the room).

An article was excluded if:

1. It only reported inferences from contexts where participants reported what they thought the emotional state of other team members was.
2. Emotional components or team communication (or both) were not the primary focus of the article, or the setting was outside of the OR or the main focus was provider-patient communication.

### Selection Process

The initial list of article abstracts was screened by one researcher (H.L.) and categorised into ‘include’, ‘unsure’ or ‘exclude’ using the selection criteria outlined above. Next, H.L. assigned each abstract a number, and, using a random number generator, randomly selected 5% of abstracts (102 of 2,047 abstracts). These were divided into three sets of 34, each to be checked by one of the other researchers (J.W., R.W-K. or A.M.) by independently determining the inclusion or exclusion status of the articles. The authors agreed on the selection status of 97% (99 out of 102) of the abstracts. With the abstracts categorised as ‘unsure’, the authors reached consensus through discussion and through reading the full article where necessary. We searched the reference lists of included articles and those initially classified as ‘unsure’ for potentially relevant articles.

Information was extracted from the included articles and entered into two spreadsheets, one for qualitative studies, and one for quantitative and mixed method studies (see Tables 1 and 2). The Sample, Phenomenon of Interest, Design, Evaluation, Research type (SPIDER) framework [23] was used for qualitative studies and the Population, Intervention, Comparison, Outcome, Time (PICOR) framework [24] for quantitative and mixed methods studies.

**Table 1.** Summary of quantitative studies included in the review.

Study	Population	Intervention	Comparison	Outcome	Time	MMAT score (range 0–4)
**THEME 1: Emotional experiences in the OR and their contributors**						
Nurok et al. [35]	Teams of staff in 4 thoracic surgery OR: nurses, anaesthetists, surgeons, technicians *Sample size not reported	Two 90-min team training sessions with discussions of team skills, communication, leadership, and opportunities to role play communication strategies	No control group	No significant change in emotional climate (degree of engagement and tenseness between staff) after intervention. Communication and team skill scores improved post-intervention but the effect was not sustained.	Pre-intervention, post-intervention and sustaining period measures	3
Katz et al. [36]	*N* = 76 anaesthesiology residents	To determine how surgeon’s incivility behaviour influenced residents’ performance in a simulated scenario Experimental group was randomised to ‘rude’ surgeon: impatient but not overly intimidating (i.e., no verbal or physical abuse)	Identical to experimental group, except the surgeon was courteous and interactions were straightforward	Self-reported data 65% experimental group felt surgeon’s behaviour negatively affected their performance compared to 25% in control group (*p* = 0.009) Objective measures Experimental group scored lower on technical (e.g., medical decision making, diagnosis) and non-technical measures e.g., vigilance, communication, teamwork. 91.2% control group rated as performing at expected level, but only 63.6% experimental group did (*p* = 0.009). Finding also confirmed by regression: exposure to incivility was the only predictor of performance (*p* = 0.007)	One-time assessment after the simulated scenario	3

**Table 2.** Summary of Qualitative Studies included in the Review.

Study	Sample		Phenomenon of Interest	Design	Evaluation	Research type	MMAT score (range 0–4)	
**THEME 1: Emotional experiences in the OR and their contributors**								
Armour et al. [27]	*N* = 9 anaesthetic nurses who participated in at least one Interprofessional Simulation Education (IPSE) team training courses in communication		To explore anaesthetic nurses’ perceptions of learning through IPSE	Qualitative semi-structured face-to-face group interviews, audiotaped	Themes: (1) Learning during IPSE Nurses reported feeling vulnerable when performing in an unfamiliar environment where they may face criticism (e.g., don’t know where things are during simulation), despite the ‘safe’ learning environment. Also when medical staff were asked to do nursing tasks during simulation, nurses felt annoyed and that restricted their critical thinking. (2) Interprofessional team communication ‘Dream team’ communication where doctors are respectful and everybody can speak up, which differed from reality. Communication frustrations and lower confidence reported when nurses failed to engage with the team were overlooked and excluded. (3)Transferring new learnings into practice Barriers: Hierarchical culture of OR, daily routine of busy OR, staffs’ reluctance to change or engage fully in team communication	Interview study		3
Wetzel et al. [28]	*N* = 16 surgeons		To investigate surgeons’ perceptions of surgical stress, highlight key stressors, and their impact on performance, and identify coping strategies	One researcher conducted 16 semi-structured individual interviews	Widespread tendency for surgeons to say that stress was not a problem for them Key stressors include unexpected surgical complications, emergency cases, equipment problems, teamwork problems, distractions Surgeons’ responses to high stress include feeling anxiety, anger, frustration, irritation, pressured, tendency to rush, unable to think clearly, change in communication e.g., reduce exchange of information with team members to a minimum, short-tempered Coping strategies include try not to show own stress to reduce tension among the team, communicate clearly, be assertive	Interview study		2
Skramm et al. [29]	*N* = 11 OR nurses		To explore how OR nurses experience communication and teamwork in the OR, particularly in areas such as acting assertively, exchanging information and coordinating with others	Qualitative semi-structured interviews with individual participants recorded electronically	Themes: (1) Hierarchical communication: Surgeons define who may speak and who may not. Experienced nurses found it easier to speak up, ‘daunting’ for new nurses. (2) Incivility: Surgeons restricted their communication to barking commands. Nurses ‘prepared to accept unpleasant communication to maintain a good atmosphere in the OR’. (3) Surgeons’ lack of preparation and sudden changes in routines and preferences caused stress and frustration in nurses, and negative communication in the whole team	Interview study		4
Chrouser et al. [30]	*N* = 42 urology residency students		To explore students’ observations of disruptive behavior during surgery and their perspectives on the ideal intraoperative working environment	Qualitative interviews with individual participants recorded on field notes	Themes: (1) Characteristics of intraoperative disruptive behaviour: 98% students witnessed or experienced at least one disruptive behaviour, most commonly from a surgeon, nonphysical/verbal (e.g., yelling, cursing, berating) more common than physical (e.g., throwing instruments). Women described a wider variety of disruptive behaviours than men. (2) Disruptive behaviour often occurred in the context of strong emotions, most commonly, feeling frustrated (3) Negative consequences on communication and decreased psychological safety: students feeling scared, increased tension and decreased morale amongst other OR team members, patient care affected (4) Ideal OR: calm and respectful, members feel appreciated, building good relationships within team, minimising behaviours that cause emotional distress	Interview study		3
Higgins &MacIntosh. [31]	*N* = 10 OR nurses	To explore nurses’ perceptions of the effects of physician-perpetrated abuse on their health and their ability to provide patient care		Researchers conducted and audio-recorded 10 individual interviews	Majority of abuse reported by nurses were psychological e.g., being belittled, ridiculed, yelled or sworn at; some were physical Abuse was more likely to occur when nurses and physicians work closely for prolonged periods of time (e.g., nurses were safe targets to release frustrations on when problems occurred); hierarchical culture (e.g., not feeling fully included in the OR team, excluded from physicians’ conversations, bear the brunt of physician’s bad mood, complaints of abuse went unheard); nurses new to the OR (e.g., physicians feed off nurses’ nervousness and do things that make nurses feel uncomfortable) Effects of abuse on nurses include feeling worthless, lacking confidence, inadequate, disrespected, belittled, effects on physical health, personal life, poor concentrations threatened patient safety	Interview study		2
Dossett et al. [32]	*N* = 30 women surgeons	To describe and understand interprofessional conflict involving women surgeons: context surrounding the conflict, implications and how women surgeons manage the conflicts		Qualitative semi-structured interviews with individual participants recorded electronically	Themes: (1)Context: Often due to communication breakdowns and women surgeons’ responses to perceived performance issue of interprofessional team member (often also women) was described as ‘sarcastic’ or ‘mean’ but those responses were acceptable if came from male surgeons e.g., assertive direction (2) Implications: Emotional (feelings of self-doubt, frustration, depression, devastation, humiliation, anxiety), professional (perceived harm in reputation, career or promotion) and patient outcomes (delays, avoiding interaction with staff due to fear of conflict, tense and ineffective communication). Women surgeons ultimately frustrated having to conform gender over professional norms. (3) Strategies: Relationship management (e.g., gauging the emotional responses of others and recalibrating own actions), rapport building, social support	Interview study		4
Rogers et al. [33]	*N* = 31 circulating nurses, *n* = 35 surgeons	To evaluate conflict transformation in OR teams, identifying behaviours associated with conflicts, behaviours that caused conflict to shift and progress, and to clarify the appropriate approach for an educational conflict management programme for surgeons		Researchers conducted 40 semi-structured discipline-specific (all nurses or all surgeons) focus groups with 3–9 participants in each group, audio-recorded	Analysis identified examples of task-related conflicts (e.g., equipment needs and scheduling), and relationship-related conflicts (e.g., bad moods or attitudes, rudeness, outbursts, blaming, insulting or threatening). Both can lead to positive (e.g., increased staff satisfaction) or negative outcomes (e.g., feeling incompetent, misery, decrease willingness to communicate with the team) Misattribution (e.g., personal attack, blaming) and harsh language lead to strong negative emotions and can cause conflict to progress and shift to negative outcomes. Negative emotions exacerbate negative and decrease positive outcomes. Solutions: Constrain negative emotions, teach alternative effective behaviours or relationship-rehabilitating behaviours e.g., apologising, to avoid conflict escalation.	Focus group study		3
Grade et al. [34]	*N* = 11 attending surgeons, *n* = 10 attending anaesthesiologists, *n* = 11 resident surgeons, *n* = 4 resident anaesthesiologists, *n* = 9 medical students, *n* = 9 nurses and surgical technicians	To explore contributors and barriers to optimal communication across different roles in the OR		Researchers conducted 54 one-on-one semi-structured interviews and 3 focus groups	Contributors towards effective communication: (1) Team familiarity e.g., know each other’s OR habits and equipment preferences (2) Regular focused communication about the surgery e.g., surgeon’s verbal description of maneuvers and surgical plan, updates, complications. Respondents reported feeling excluded and disengaged without these communication (3) Surgeon’s mood and tone - 60% respondents felt this alter the effectiveness of communication. Respondents more comfortable with surgeons who maintained a positive tone and attitude (4) Formal communication e.g., standardised checklists, time-outs Ineffective communication: (1) Unclear expectations for each role in the OR (2) Hierarchy and social structure	Interview and focus group study		3

We used the Mixed Methods Appraisal Tool (MMAT) [25] to assess the methodological quality of each of the empirical studies included in the review. The MMAT has a set of guidelines for each type of study (i.e., qualitative, quantitative RCT, quantitative non-randomised, quantitative descriptive, and mixed methods). Each set of guidelines has four criteria for determining how well the study was conducted to answer the research question. For example, one of the criteria for a qualitative study would be: was appropriate consideration given to how the findings relate to the context? And for a quantitative RCT: was there a clear description of the randomisation process? The total score ranged from zero to four, where a higher score represents higher quality.

A narrative synthesis of the included articles was conducted following the guidelines described in Popay et al. (2006) [26], where the data from included articles was explored, and patterns and relationships sought between the different studies.

The analysis was led by H.L. with input from the full research team at monthly intervals, and through email circulation of each component of article selection, article appraisal and analysis. The research team agreed on all aspects of the analysis and narrative synthesis, with disagreements resolved through discussion and review of the data.

## Results

**Figure 1.**
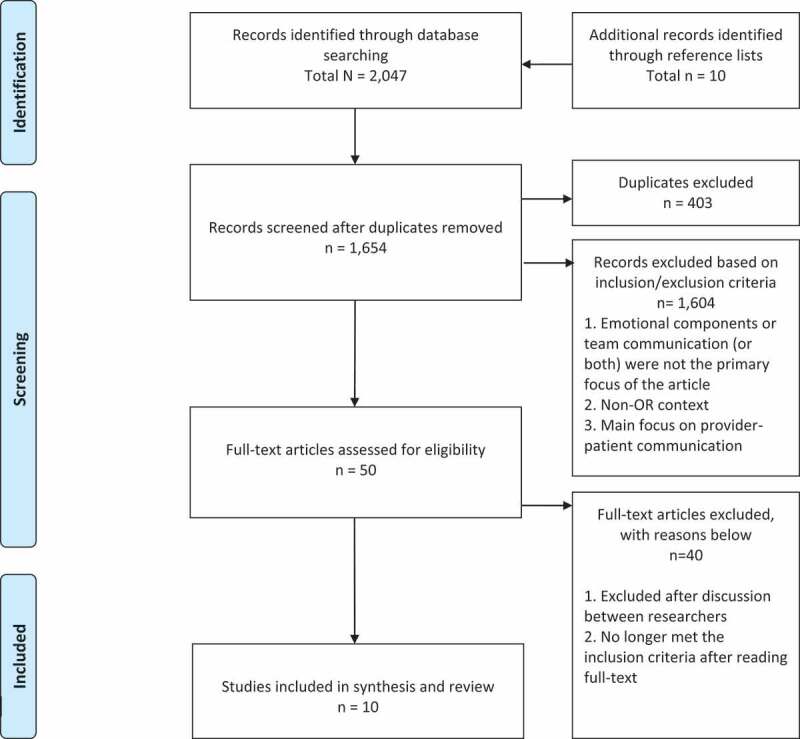
PRISMA flow diagram of the search process.

A total of 10 articles met the inclusion criteria for the review. Figure 1 shows the PRISMA flow diagram of the search process. Of the 10 articles, eight were qualitative studies and two were quantitative studies. Of the eight qualitative studies, six were interview studies [27–32], one was a focus group study [33], one was an interview and focus group study [34]. Both of the quantitative studies were interventional studies: one used a pre-post design [35] and one was a randomised controlled trial [36]. All the included studies were of reasonable methodological quality, i.e., obtained an MMAT score of two or above. Therefore, all of the studies were included in the final review and discussed in the synthesis. The included studies are summarised in Tables 1 and 2.

The included studies examined either the emotional triggers influencing communication or the emotional responses to communication in the surgical team. The studies could contribute to more than one of the identified three themes. These three themes were: (1) Emotional experiences in the OR and their contributors (10 studies); (2) Effects of emotional experiences on team communication (seven studies); and (3) Solutions to manage the emotional experiences in the OR (three studies). Studies could fall into more than one theme or sub-theme.

The first theme was the largest, and was divided into three sub-themes: The first sub-theme was range of emotions experienced in the OR (1a). The second sub-theme was hierarchical culture (1b) and how it contributed to the emotional experiences in the OR. The third sub-theme was leadership expectations (1c) and the emotional impact of perceived failings in the leader. Table 3 lists the studies included under each theme and sub-theme.

**Table 3.** Studies included under each theme and sub-theme.

Theme	Sub-theme	Studies
1: Emotional experiences in the OR and their contributors		
	1a: Range of emotions experienced in the OR	[27,28,30–32,35]
	1b: Hierarchical culture	[27,29–31,33,34]
	1c: Leadership expectations	[28,29,34,36]
2: Effects of emotional experiences on team communication	N/A	[27,29,31–34,36]
3: Solutions to manage the emotional experiences in the OR	N/A	[28,32,33]

### Theme 1: emotional experiences in the or and their contributors

#### 1a. range of emotions experienced in the OR

Six studies identified the various types of emotions experienced by health professionals in the OR. In one study, Wetzel et al. [28] outlined the sources of stress in the OR, which included unexpected surgical complications, emergency cases, time pressure, equipment problems, and interpersonal issues between team members [28]. These stress-inducing events led to a range of emotions experienced by health professionals working in the OR. Surgeons often reported feeling anxious, angry, frustrated, irritated, a sense of urgency to think and act quickly and a tendency to rush when experiencing highly stressful situations in the OR [28]. In a simulation study by Armour et al. [27], anaesthetic nurses reported feeling vulnerable working in a team where members were unfamiliar with each other, and in an environment which was different to their usual workplace. Feelings of frustration and low self-confidence were commonly reported in the OR, when some team members were excluded from decision-making, overlooked and felt unable to speak up [27]. Team members also reported feeling scared, insecure, disrespected and frustrated when exposed to incivility or disruptive behaviour [30,31] or conflicts [32] in the OR. Nurok et al [35] devised a method to assess emotional climate in the OR, using observers’ ratings of the degree of tenseness and degree of engagement of health professionals in the OR and reported that the emotional climate of the OR was mostly engaged (e.g., staff alert, interested) and appropriately tense (e.g., staff’s level of anxiety was appropriate to context) [35]. Taken together, these studies suggested that the OR can be a highly stressful working environment. Health professionals reported feeling a range of negative emotions as a result of this stressful environment.

#### 1b. Hierarchical culture

Six studies identified hierarchical culture as a major contributing factor towards these emotional experiences in the OR. A hierarchical culture was described where surgeons and senior doctors had power over trainees and other professionals (in particular, nurses). Studies have reported team members’ reluctance, difficulty and feelings of frustration experienced when speaking up to those at the top of the hierarchy [27,29,31,34]. Feelings of frustration and low self-confidence were also reported by nurses who were excluded from conversations between doctors [27,31]. Disruptive behaviours were another contributing factor towards negative emotional experiences in the OR. The disruptive behaviour identified in our included studies were unacceptable or inappropriate behaviour that could hinder teamwork, communication and psychological safety. A number of interview studies in our review reported the negative emotions experienced by health professionals that arose as a result of disruptive behaviour [27,29–31,33]. In an interview study by Higgins and MacIntosh [31], nurses were reportedly subjected to being the brunt of a surgeon’s bad mood and a ‘safe target’ to release the surgeon’s frustrations. Nurses also reported surgeons deliberately making the already anxious nurses feel uncomfortable by ‘stomping around the room’ [31]. Similarly, Chrouser et al. [30] reported that 98% of trainees had experienced surgeons’ disruptive behaviour in the OR, most commonly yelling, swearing, ‘barking’ commands, criticising, and throwing objects around. Trainees and nurses reported feeling scared and frustrated and concerned that the disruptive behaviour created tension in the room [30]. Despite this, some surgeons believed these behaviours were sometimes justified in order to quickly accomplish some task-related goals [33]. In the interview study by Chrouser et al. [30] above, trainees defended the surgeon’s disruptive behaviour and attributed it to stress and frustration.

#### 1c. Leadership and expectations

Four studies pointed out how the emotional state of the leader and that of the team members can affect each other in team communication. Three out of four studies have suggested that the tone and attitude of the leader (most often the surgeon) set the mood in the OR and directly influenced communication effectiveness [28,34] and job performance in the OR [36]. In the study by Grade et al. [34], OR members felt more comfortable when the senior surgeon maintained a positive tone and attitude throughout the procedure [34]. In a simulation study by Katz et al. [36], anaesthesiology residents scored lower in all performance measures when working under a surgeon portrayed as ‘impatient’ compared to a ‘courteous’ surgeon [36]. The surgeons in the study by Wetzel et al. [28] reported that they had to make an effort not to show their own stress, in order to reduce tension among the surgical team members [28].

Leaders were also expected to communicate preferences, routines and information about the procedure to all team members. Skramm et al. [29] found that surgeons who did not clearly communicate their preferred instruments or suddenly wanted different instruments created stress, caused frustrations and poor communication among team members [29]. Similarly, Grade et al. [34] reported that when nurses and technicians were unable to tailor the equipment needs of the senior surgeon, communication ‘fell apart’. In addition, anaesthetists felt excluded and disengaged when the senior surgeon did not communicate to them the plans of the procedures and any updates during the procedure, which resulted in confusion and communication failures among team members [34].

Overall, these studies suggested that team members expect leaders of surgical teams to have good control of their own emotions, remain positive and clearly communicate their preferences and plans to all team members throughout the entire procedure. Negative emotions could arise if these expectations were not met.

To summarise Theme 1, the OR is an emotionally charged environment. The team expectations of the leader, coupled with the hierarchical culture of the OR, can create negative emotions and tension between team members which can compromise interpersonal dynamics as well as surgical team performance.

### Theme 2: effects of emotional experiences on team communication

Seven studies reported the effects of negative emotional experiences on team communication in the OR. Within a hierarchical OR culture, the power difference between doctors and nurses influenced interpersonal communication, resulting in reluctance, difficulty and feelings of frustration when speaking up to those at the top of the hierarchy [27,29,31,34]. In an interview study by Skramm et al. [29], nurses reported that some surgeons dictate who may speak in the OR and who may not. Other surgeons reduced their communication to ‘barking’ commands and communicated their preferences at the last minute, which nurses found frustrating and daunting [29]. Some nurses indicated they were ‘prepared to accept unpleasant communication (from the surgeon) to maintain a good atmosphere in the OR’ [29] suggesting that incivility, and its consequences on team communication and function may go unchecked. After exposure to incivility, trainees were more reluctant to communicate with the surgeon [36] and nurses reported withdrawing communication and avoiding eye contact with the surgeon [31]. Similarly, other interview studies noted that health professionals involved in conflicts were more likely to avoid further interactions with each other [32,33].

Taken together, these studies in Theme 2 describe how a hierarchical culture can inhibit nurses and trainees from speaking up and voicing their concerns [27,29,31]. The OR team may be unclear about the plan for the procedure or needing an update on progress, but feel unable to seek clarification [34], compromising the ability of the team to prepare for anticipated events and respond to changes in the patient’s condition. Previous negative interactions can result in team members withdrawing from communicating or interacting with other members of the surgical team, potentially limiting their contribution to ensuring the safety of the patient.

### Theme 3: Solutions to manage the emotional experiences in the OR

Three studies reported on participant suggestions or strategies for managing stressful situations in the OR. In the interview study by Wetzel et al [28], surgeons described coping strategies in stressful situations. These included (1) recognising the signs that they were stressed (e.g., heart pounding, clouded judgment); (2) stopping what they were doing and standing back; and (3) regaining control of self and the situation. With regard to regaining self-control, surgeons described techniques such as physical relaxation, distancing, self-talk, and trying not to show stress themselves in order to avoid creating stress in the team. To regain control over the situation, surgeons would pause to reassess the situation, make a decision, then plan and prepare for the next stage [28].

Two interview studies focused on how to manage conflicts provoked by stressful situations in the OR which could hinder effective communication. Rogers et al. [33] analysed conflicts in the OR and identified a set of behaviours that caused conflicts to progress or shift to negative consequences on communication and team dynamics. These behaviours included misattribution (e.g., blaming) and the use of harsh language (e.g., threats, insults, yelling, profanity). According to Rogers et al. [33], these behaviours could induce negative emotions and exacerbate conflict. Surgeons could potentially be educated on conflict management techniques such as how to constrain negative emotions and remain calm, and using alternative behaviours (e.g., apologising or other relationship rehabilitating behaviours) [33]. Dossett et al. [32] interviewed women surgeons who had been previously involved in conflicts with staff from a different discipline. They reported using strategies such as rapport building, relationship management techniques (e.g., gauging the emotional responses of others and recalibrating own actions based on those responses), and seeking out social support (e.g., talking about shared experiences with colleagues) to navigate the conflicts [32].

Overall, studies included in Theme 3 focused on senior doctors’ own awareness of stressors that can affect their performance and potential coping strategies, responses to stress or frustration that may escalate conflicts within the OR team and potential educational interventions to learn to better manage conflict.

## Discussion

In this scoping review we identified ten studies that reported on the emotional aspects of communication between health professionals working in the OR. These studies fell into three main themes: (1) Emotional experiences in the OR and their contributors; (2) Effects of emotional experiences on team communication; and (3) Solutions to manage the emotional experiences in the OR.

Our review highlights the emotionally charged environment in which OR teams work and the emotional responses to the traditional hierarchy. Failure of leaders to meet the team expectations of appropriate and timely communication may lead to team feelings of frustration and stress. The hierarchical culture can inhibit nurses and trainees from speaking up and voicing their concerns, or seek clarification of the plan, compromising the team’s ability to prepare and plan for the procedure. Team members may disengage from patient care as a result of previous negative interactions. Surgical views on managing this emotional undercurrent included managing their own stress and learning better conflict management skills.

### Relation to broader literature

The included studies reported a variety of emotional triggers to communication in the OR such as feelings of anxiety, anger, irritation and frustration. Stressful events (e.g., unexpected surgical complications, time pressures, equipment and interpersonal issues) triggered these negative emotional experiences, which then reduced the quality of communication between team members and, as a result, triggered additional stress in the team members responding to the communication. This stress then led to negative emotional responses to the suboptimal communication, such as feeling disrespected, scared, insecure, frustrated. Thus, a vicious cycle was created of ineffective team communication. This finding is in line with other studies in the healthcare literature. For example, in the nursing literature, Thornby [37] explained that previous negative experiences in communication with a colleague created stress, anxiety and irritation in subsequent communication encounters, leading to ‘flight’ (e.g., becoming silent, ignoring) or ‘fight’ responses (e.g., sarcasm, angry responses), resulting in a continuing cycle of ineffective communication [37]. Ineffective communication can lead to delays and errors in patient care, which can compromise patient safety [20,38]. Our study, with its focus on the OR context, adds to the existing literature through exploring the subsequent impact on team communication in the OR when health professionals experience negative emotions during the course of their work. Incivility or disruptive behaviour is more likely to happen as a result of the high levels of stress that health professionals experienced, and stress is a feature of patient care in the OR environment. Incivility or disruptive behaviour and its damaging effect on interpersonal relationships have been reported in other acute care contexts [39–41] as well as the OR [42–44].

Conflicts and disagreements can arise more easily in stressful situations and this has adverse implications on team communication. Interestingly, only three studies in the present review examined strategies used by health professionals to deal with conflicts with colleagues in the OR [28,32,33]. Rogers et al. [33] described the type of behaviours that could exacerbate conflicts, to contribute to the development of a conflict management intervention for surgeons in the OR. Sinskey et al. [20] went a step further by conducting a review on the conflict management literature and identified the phases of conflict and described the different types of conflict management styles and techniques that health professionals could use [20]. According to Sinskey et al. [20], health professionals must first recognise the phases of conflict, then identify and apply the most appropriate conflict management strategy to use in the given context. Sinskey et al. [20] suggested three conflict management strategies in the OR that health professionals could use: (1) acknowledging and managing own emotions before reacting; (2) seeing beyond the other person’s emotions and trying to understand their perspectives, reasoning and concerns; and (3) aligning interests and emphasising common goals to identify alternative options and solutions. In another study looking at surgeon’s stress and coping strategies, Arora et al. [45] suggested that a stress-management intervention for surgeons should include the following components: (1) acknowledging stress and its impact on performance; (2) cognitive training to teach surgeons to remain calm and focused in a stressful situation; (3) practising stress management skills in a safe and controlled environment, such as simulation; (4) training as a team together; and (5) providing individualised feedback and self-reflection, for example, post-simulation debriefing. The review by Sinskey et al. [20], Arora et al. [45], along with the studies in the present review, offer valuable insights into why conflicts occur and recommended strategies that could potentially be useful in the OR. However, the effectiveness of these is yet to be determined. This represents a gap in the literature that future studies could address.

### Limitations

The topic *emotional components* of communication included a broad range of studies across different fields. Some researchers might have described the phenomenon using very specific terms, such as frustration, aggression, while others might have discussed ideas relevant to the topic without actually using the word *emotion* at all, or any of its synonyms. Therefore, some potentially relevant articles may have been missed.

All 10 studies in the present review have examined negative emotional experiences in the OR. It is possible that there is a literature bias in reporting negative emotional experiences and taking for granted the positive emotional experiences that happen on a regular basis. Other studies suggested that proactively establishing rapport in ad hoc surgical teams [10] and expressing gratitude and appreciation for each other’s work [46] may create a more positive atmosphere in the OR. Future research should also consider what contributes to positive emotions in the OR and how to increase positive emotional experiences in the OR.

## Conclusion

In this review, we identified 10 studies that described different types of emotional triggers and emotional responses to communication, and examined the ways that these negative emotions can affect communication in a surgical team. The hierarchical culture and expectations of being a leader in the OR contributed to these negative emotional experiences. These negative emotional experiences had detrimental effects on team communication, for example, health professionals withdrawing or avoiding communication with each other, which could compromise patient safety. Only three studies explored ways to reduce negative emotional experiences in the OR and none provided evidence of effectiveness. Thus, these are clearly areas warranting future research.
